# Risk perception, barriers, and working safely with silica dust in construction: a psychological network approach

**DOI:** 10.1186/s12889-025-23347-2

**Published:** 2025-07-03

**Authors:** Tom Jansen, Myrthe von den Benken, Gabriela Lunansky, Evi van Moll, Marre Lammers

**Affiliations:** 1 Centre for Environmental Safety and Security, Division Environment and Safety, Dutch National Institute for Public Health and the Environment (RIVM), Bilthoven, the Netherlands; 2 Department of Epidemiology & Data Science, Amsterdam Public Health research institute (APH), Amsterdam University Medical Center, Amsterdam, the Netherlands

**Keywords:** Risk perception, Occupational health and safety, Construction, Silica dust, Preventive measures, Risk communication, Behaviour, Psychological network analysis

## Abstract

**Background:**

In the construction industry workers are frequently exposed to hazardous substances. To explore and describe construction workers’ barriers and motives to (not) work safely with hazardous substances, we examined their perspectives on the health risks, perceived barriers, and intention to use preventive measures with regard to silica dust. Specifically, we studied perspectives on the use of face masks, dust collection on power tools, and using a vacuum cleaner instead of a broom.

**Method:**

Semi-structured interviews (*n* = 13) and a pen and paper survey (*n* = 187) were administered on construction and training sites. Only executive workers could participate in the study. We approached the behaviour of using specific preventive measures as an emergent property of a complex network of interacting psychological variables. To analyse the structure of these ‘behavioural decision networks’ we applied a psychological network.

**Results:**

Through the exploratory semi-structured interviews, we identified themes relevant for our survey, like perceived exposure, risk being considered as part of the job, and perceived barriers like time, effort, and properties of the work environment. Construction workers were generally aware their health is at risk due to occupational exposure to silica dust. At the same time, they are not overly concerned about that risk. Network analysis suggests that concern does play a moderate role in the behavioural decision networks, suggesting that a lack of concern may encourage unsafe behaviour. Construction workers’ level of automaticity to use specific preventive measures was relatively low. Barriers to use preventive measures such as time and effort play a relatively key role in the networks. A general intention to work safely hardly played any role in the networks, while a specific intention to use preventive measures played a more prominent role. Age and work experience did not play a role in the network. Non-parametric tests and descriptive comparison of networks suggest differences in for example the relative importance of specific variables.

**Conclusions:**

For two preventive measures, different variables may be more successful intervention points to foster safe work. Increasing levels of concern, improving automaticity of use, addressing specific intention to use preventive measures in risk communication, and offering preventive measures at time and location where relevant tasks are performed, are discussed as possible intervention points to foster working safely with silica dust in construction. Future studies should further substantiate these findings.

**Supplementary Information:**

The online version contains supplementary material available at 10.1186/s12889-025-23347-2.

## Background

In the Netherlands, it is estimated that each year around three thousand people die due to exposure to hazardous substances at the workplace [1, 2]. An even greater number of people become ill. Construction is an industry in which workers are frequently exposed to hazardous substances. It is broadly acknowledged there is a need for practical knowledge that can help prevent and reduce occupational diseases from exposure to hazardous substances. Effective prevention partly lies with the decisions of construction workers to comply with or use preventive and protective measures. To form a better understanding of construction workers’ barriers and motives to (not) work safely with hazardous substances, we examined their perspectives on the health risks, the barriers they experience to use preventive measures, their intention to use preventive measures, and their intention to work safe in general with regard to Respirable Crystalline Silica (CAS 14808–60-7), also known as silica dust.

Several health behavioural models and theories can be used as a starting point for studying preventive behaviours by construction workers. Three prominent examples are the Theory of Planned Behaviour (TPB, [3, 4]), the Health Belief Model (HBM, [5]) and the Protection Motivation Theory (PMT, [6]). Many core concepts in these models are similar. For example, these models all include perceived vulnerability, severity of consequences and self-efficacy as core concepts to explain behaviour. However, some differences exist. In the current study we use the PMT as a starting point for our research because it is the only model that also accounts for perspectives on the perceived effectiveness of risk reduction measures (i.e. response efficacy). In their meta-analysis on the PMT, Floyd and colleagues concluded that PMT can provide useful starting points for developing interventions to promote preventive behaviours [6]. The PMT has been applied to study preventive behaviours with regard to for example unwanted pregnancy and sunburn, and more recently COVID-19, and occupational hazards faced by emergency ward nurses [7–10]. In the current study, we approach PMT as a descriptive model and extend the core concepts (perceived vulnerability, perceived severity, self-efficacy, and response efficacy) with context specific factors relevant to risk perception of chemical substances in construction. We elaborate on these factors in more detail below.

Risk perception, as used here, is considered a psychological process that comprises of complex interactions among values, attitudes, (mis)beliefs and feelings associated with the risk at hand. At the same time, risk perception also comprises evaluative outcomes such as perceived severity or perceived (personal) vulnerability. Risk perception hereby holds cognitive and affective elements that may serve as drivers and barriers for working safely [11, 12]. Knowledge on key aspects in workers’ risk perception of chemical substances may thus hold key insights that can help to prevent and reduce substance-related occupational diseases. For example, research has shown that a lack of experience with negative consequences of unsafe work can cause someone to believe that they themselves are less likely to experience a negative event ([13, 14], see also [15]). Additionally, studies have shown that when workers underestimate occupational risks, they are more likely to conduct risky behaviours [16, 17].

Most studies on hazard and risk perception and the use of preventive measures in the construction industry study perception of safety risks, like falling from height, cuts and wounds and handling heavy loads ([13], see e.g., [18–21]). Zhang and Fang [20] for example studied the reasons why Chinese scaffolders do not use fall harnesses. They concluded that underestimating the risk of not using safety harnesses, the inconvenience and discomfort of using safety harnesses, negative pressures from people in exemplary positions (e.g. foremen and safety officers), and lack of safety lines are causes of scaffolders deciding not to use safety harnesses.

Few studies have examined workers'perceptions of occupational health risks from chemical substances in the workplace. Studies that do, provide several leads on how risk perception and use of preventive measures are associated. For example, Antonucci et al. [22] showed in a sample of Italian construction workers that risk perception is higher regarding hazards that can cause immediate injury, as opposed to those that can cause occupational illnesses. If workers underestimated the probability of experiencing adverse effects from exposure, this could lead to ineffective use of personal protective equipment (PPE) and may even foster risky behaviour [23, 24].

In a qualitative study, Hambach and colleagues [25] explored worker perceptions of chemical risks, to find leads for a workplace health program. Their sample consisted of workers who worked in paint production, cleaning and maintenance, or in production of surface-active agents. Hambach and colleagues found that workers partly rely on their senses to assess risks (see also [26–28]), often accept risks associated with their job, and mistrust prevention advisors. In another study by Pettersson-Strömbäck and colleagues [29] workers from the plastic industry were interviewed to study their mental models (i.e. their collections of beliefs, attitudes, feelings, etc. on a particular topic) of exposure to chemical substances. These mental models were compared to actual exposure data. The results showed that workers tend to estimate exposure based on their work activities, which generally resulted in an underestimation of their exposure compared to what the actual exposure data showed. In a study on the relationship between noise hazard perception of miners and their use of earplugs, Shkembi, Smith, and Neitzel [24] also found that about one in three workers underestimated their exposure to noise when their true exposure was hazardous. While not statistically significant, misperception of exposure to hazardous noise was associated with higher odds of not using earplugs.

Remoundou et al. [30] concluded that workers are more likely to adopt self-protective behaviours if they perceive their health has been negatively affected by the use of pesticides. In another study also providing insight in the use of protective measures, Stege and colleagues [31] applied the mental models approach (see also [29, 32]). They found that mental models of (the risks of) particulate matter of road workers differed from the mental models of scientific experts on the topic. Results further showed that while workers are aware of the existence of particulate matter and ‘reduction methods,’ their knowledge is often incomplete, and they do not protect themselves consistently against particulate matter.

Further insights from public risk perception of chemicals provide additional leads on how risk perception factors may serve as drivers and barriers for working safely. For example, studies have shown that non- experts tend to use values, norms, attitudes and psychometric aspects such as perceived familiarity with the risk, perceived controllability of exposure, and the delayed occurrence of adverse effects to evaluate risks [26, 28, 33–37].

Taken together, the above-described literature suggests that a combination of many factors likely influences construction workers’ preventive behaviour with regard to occupational exposure to chemicals. An integrative study looking at a combination of these factors can help further efforts in the prevention and reduction of substance-related occupational diseases.

### Current study

**Fig. 1:**
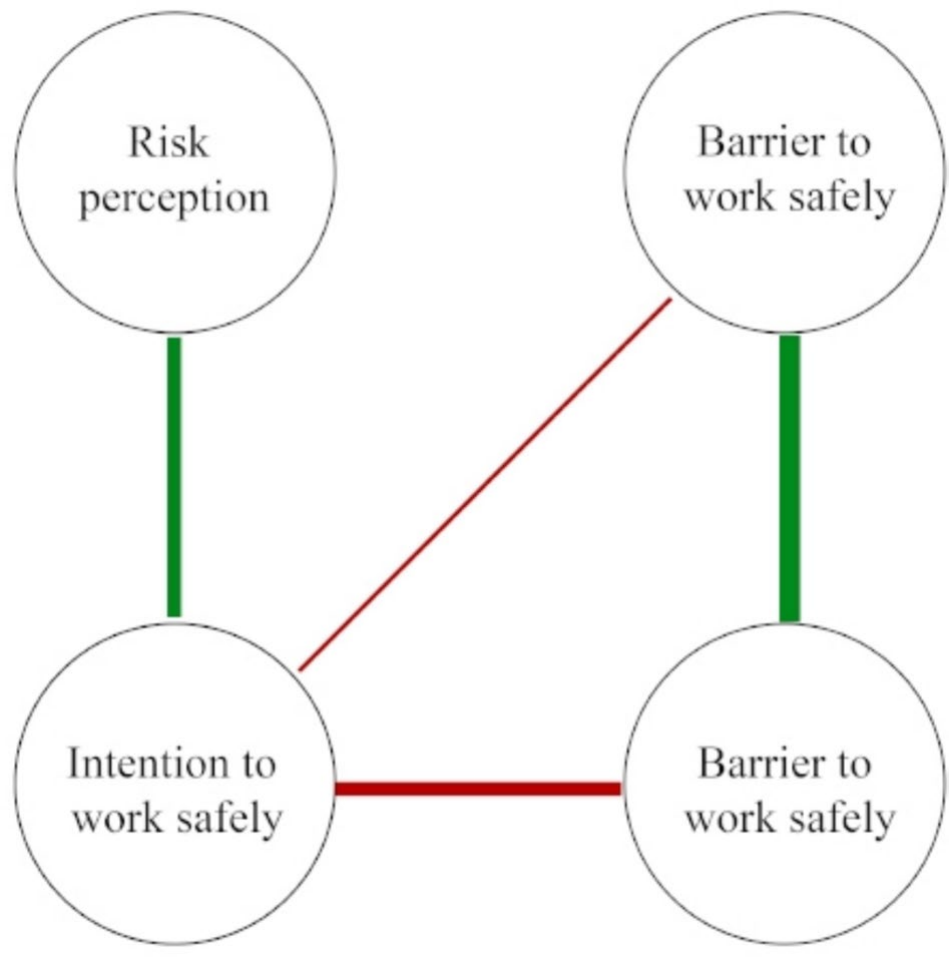
Schematic representation of a network topology. *Note*. For illustrative purposes only. Red lines (i.e. ‘edges’) indicate a negative relationship, indicating that a higher value of one variable (i.e. ‘node’) is associated with a lower value of the other variable (and vice versa). For example, in this schematic representation a higher perceived barrier to work safely is associated with a lower intention work safely. Green lines indicate a positive relationship indicating that a higher value of one variable (i.e. ‘node’) is associated with a higher value of the other variable. Thicker lines indicate a stronger relationship. Unconnected nodes are independent from other nodes, taking into account all the other nodes in the network

The current study presents an explorative and descriptive account of the risk perception of constructions workers and the perceived drivers and barriers towards the use of preventive measures related to exposure to chemical substances in the construction industry. First, we conducted exploratory qualitative interviews to identify relevant themes and ensure content validity and relevance. In our subsequent quantitative study we follow the Causal Attitude Network (CAN) model [38] to describe construction workers’ risk perceptions and barriers to working safely. Recent studies have demonstrated that the CAN model can help improve our understanding of risk perception, preventive behaviours and can help design behavioural interventions [39–41]. The CAN model conceptualizes attitudes (e.g. an overall evaluation of an event or behaviour) as psychological networks of evaluative elements (e.g. beliefs, feelings, and behaviours) that interact causally and can be understood through a network topology. This means that beliefs and feelings that are more closely related will be arranged more closely in the network structure. Complex systems like the CAN have emergent properties that are not explained by understanding the individual elements of the system [42, 43]. Following this, we approach the actual use of preventive measures (i.e. safe work behaviour) as an emergent property of a system of interacting (psychological) variables (see also [44, 45]). In this conceptualization, specific patterns of interacting variables within the system (e.g. risk perceptions, perceived barriers, intentions) give rise to (un)safe work behaviour. These patterns can be presented in a network topology. Figure 1 presents a schematic representation of a network topology. For this study we will refer to the networks of interaction variables as behavioural decision networks.

We investigate how risk perception, perceived drivers and barriers are related to the (intention to) use three specific types of preventive measures: using a face mask, using dust collection on power tools, and using a vacuum cleaner instead of a broom when cleaning to prevent exposure to silica dust. Second, we investigate whether differences can be observed between these three types of preventive measures.

In Europe and the Netherlands, silica dust is considered one of the most prominent health hazards for construction workers [46, 47]. Silica dust can cause severe lung problems and is classified by the IARC as a group 1 carcinogen, meaning there is definite proof that it can cause cancer in humans [48]. It is estimated that in the European Union around 5 million workers are exposed to crystalline silica dust [47]. To our knowledge the current study is one of the first studies (cf. [49]) on psychological drivers and barriers and the use of protective behaviours related to silica dust. It is also the first study to focus on safe behaviour in the construction industry using a complexity perspective.

## Methods

We recruited construction companies and visited construction sites to administrate (voluntary) semi-structured interviews. Based on the findings from the interviews and the literature presented in the background section of this paper, we developed a questionnaire and administered it to construction workers. We did this face to face at construction sites of the participating companies. The procedure is described in more detail below.

### Recruitment and participant criteria

Recruitment of companies was done through a non-profit knowledge and advice centre for the construction and infrastructure sector (Volandis) and through a public appeal to safety experts working at construction companies to contribute to a study about ‘risk perception in the construction industry.’ After companies agreed to contribute, researchers accompanied safety experts to construction sites to recruit workers on site. Only executive workers such as carpenters, ground workers and electricians were eligible to participate in the study. Workers were informed that participation to the study was anonymous and on a voluntary basis. Workers were eligible to participate in the study if they were 16 years or older, and were exposed to silica dust through their own work activities (e.g. cutting, sawing, grinding, and/or drilling bricks, concrete, or cement) or could be exposed through the work activities of others (e.g. through silica dust that in general is present at the construction site or has become airborne due to sweeping a work site). All participants provided spoken or written informed consent before participating in the study.

### Exploratory qualitative interviews

Prior to the quantitative survey, 13 exploratory semi-structured interviews were conducted to identify relevant themes for the questionnaire and ensure the questionnaire’s validity and relevance to participants.. All participants were male. Mean age was 42.9 years; mean years of work experience was 23.5 years. All participants had a lower or middle vocational technical degree. All participants had a Dutch cultural background, except for one who identified as Moroccan-Dutch. Interviews generally lasted between 20 and 30 min and followed a semi-structured protocol that covered core concepts from the Protection Motivation Theory [6, 50]. Questions (e.g. “Do you personally encounter situations in which you come into contact with silica dust?,” “Do you think that you could develop serious lung problems from exposure to silica dust?”,” and “Do you know what you can to prevent inhaling silica dust?”) aimed to elicit relevant beliefs and feelings associated with silica dust, perceived vulnerability and severity, and response and self-efficacy. After the interview, participants were debriefed and were provided with information on who to contact in case the interview caused future health concerns.

The audio recordings of the interviews were transcribed verbatim and were analysed thematically using Atlas.ti (version 7). Following Friese [51], TJ and MvdB identified the themes iteratively. Contents of segments on which the two coders disagreed were discussed and themes were refined. Several themes emerged from the interviews, including perception of exposure, the normalization of risk of silica dust as “part of the job,” and perceived barriers to preventive behaviours such as time, effort, and properties of the work environment. To illustrate these themes, a few example quotations from the interviews are provided in Table 1. Identified themes were used directly to inform item development for the questionnaire, which is discussed in more detail in the *main study and measures* section below.

**Table 1:** Example themes and quotes identified in the interviews

Theme	Quotation
Perceived exposure	“*Yeah, I wouldn’t use a mask for just one hole, but if I have to drill a lot in a day, you know, then I will.* (respondent T3)”
Risk of silica dust is “part of the job”	“*You know, I’m not going to worry about that because if I have to worry about that, I might as well leave construction.* (respondent T7)”
	“*It’s just part of your job. Look, you can get sick from anything. If it’s not from quartz dust, it’ll be from something else.* (respondent M8)”
Barrier: Time	“*It depends a little on the workload, or rather the time pressure, for example—does it need to be done quickly? If I have to walk 15 min to get a vacuum cleaner, then the hole won’t really be drilled clean* (respondent M2)”
Barrier: Properties of the work environment	“*It's often those smaller corners or the more difficult spots that you can hardly reach otherwise—then you take it off.* (respondent T1)”

### Main study and measures

To ensure data quality we aimed to design a questionnaire that was as little cognitively demanding as possible. Frequently mentioned topics in the interviews were converted to corresponding psychological constructs in the questionnaire. Based on the pilot study and identified constructs from the literature, a structured questionnaire was designed, covering factors associated with the perception of the hazard, exposure, and risk, and factors associated with the use of preventive measures. An overview of constructs and (example) items is presented the Supplementary Material 1. All items were measured using a five-point Likert scale: Completely disagree (1) – Disagree (2) – Do not disagree, do not agree (3) – Agree (4) – Completely agree (5). Before answering questions, participants were asked whether they had ever heard of silica dust. Subsequently to align ideas, a brief description of our understanding of silica dust was given. It said “*Silica dust is the name given to very small particles of silica. Silica can be found in building materials such as bricks, plasterboards, and concrete. If you work with building materials that hold silica, silica dust can come off. When we refer to silica dust in the questionnaire, we refer to these particles.”*

Risk perception was measured in terms of vulnerability in light of the PMT. Both cognitive (probability of experiencing effects) and affective (worry about experiencing adverse effects) dimensions were measured. Familiarity was measured using two items; “Working with silica dust is normal to me” and “I don’t know any different than working with silica dust” (α = 0.60). Items were summed and averaged for the analyses. For all factors associated with preventive measures we focused on three measures that can reduce or prevent inhalation of silica dust:

- using a face mask,
- using dust collection on power tools, and
- using a vacuum cleaner instead of a broom.

Data was collected separately (within subjects) for each of the three measures and included intention to use preventive measures; self-efficacy; response efficacy; automaticity of using protective equipment and barriers to using protective equipment. Constructs and (example) items are presented in Supplementary Material 1. *Automaticity of using protective equipment* was initially measured using the four item automaticity index [52] based on the Self Report Habit Index [53]. We reformulated “I start doing before realizing I’m doing it” to “I have to think about before doing it” with the aim to accommodate for lower language skills. However, based on reliability analysis this item was subsequently dropped. The remaining three items were summed and averaged. Reliability was generally good (Face mask α = 0.73; Dust collection α = 0.83; Vacuuming α = 0.83). Through the interviews we identified five barriers to using preventive measures (PM): Not having PM at hand when needed; using PM costs too much time; using PM costs more efforts than it yields; the work environment complicates the use of PM; and the use of PM complicates the work itself. All barriers were included as items in the questionnaire (example items in Supplementary Material 1).

### Data analysis of main study

All quantitative data was analysed using R (version 4.3.1). Non-parametric comparisons for the three separate preventive measures (multiple dependent groups) were conducted using the Friedman test and Wilcoxon signed-rank (WSR) test. These tests are needed to identify possible intervention points in the behavioural decision networks. For example, if a specific barrier has a central position in the network, but is not widely perceived as a barrier (i.e., it has a low mean score), it may not represent the most effective intervention point to promote preventive behaviour. Furthermore, the best intervention points to promote preventive behaviour may differ between preventive measures (e.g. using a vacuum cleaner instead of a broom may require more effort than putting on a face mask).

Subsequently, network analyses were performed. We estimated behavioural decision networks of using a face mask, using dust collection on power tools, and using a vacuum cleaner instead of a broom. In network analysis, variables included in the network topology are called ‘nodes. The nodes represent the different (psychological) factors included in the analyses (see Supplementary Material 1). The undirected connections between nodes are called ‘edges. The edges represent the mutual dependence of the nodes, given the presence of all other nodes in the network (cf. partial correlations). Thicker edges or higher ‘edge weights’ (i.e. the lines) between nodes mean they are more strongly connected (see Fig. 1) [54].

Network estimation were conducted using *mgm* (version 1.2–14) [55]. We estimated three Gaussian Graphical Models from the continuous data. The *mgm* package was used to compute and include node predictability in the network visualisations. Respondents were removed from the data set when they did not fit our respondent inclusion criterium (i.e. only executive workers) or when there were signs of, for example, straight lining in their responses. We applied multiple imputation to missing data because data were missing at random and listwise deletion is not an option in *mgm*. This way, loss of data and bias (e.g. compared to applying case wise deletion) are minimized. Networks are estimated using regularization. In this procedure, very small edges (i.e. very low partial correlations) are set to zero. As such, regularization ensures that the estimated network is sparse, and the probability of false positive edges is low. Regularization parameters were selected using cross-validation (CV).

An important metric in network analysis is *node centrality*. Central nodes in the network have more and stronger relations to the other nodes and may be important intervention targets to change the network structure. Centrality of nodes was based on node strength, which is computed based on the number and thickness of edges of each node with its neighbouring nodes.

Nodewise predictability was added as a complementary analysis [56]. Nodewise predictability represents the proportion of variance in that node that can be explained by its connected neighbouring nodes. It can help identify secondary intervention targets within the network (e.g. when the primary variable of interest cannot easily be intervened on). Nodewise predictability is calculated in terms of explained variance (R^2^).

Analyses of stability and accuracy of the networks were performed using *Bootnet* [57]. To assess stability and accuracy, bootstrapped confidence intervals (CI) of edge weights were estimated. As data was regularized, non-parametric bootstrap was applied [57, 58]. The correlational stability coefficient (CSC) was calculated to assess stability of the centrality indices (strength). The CSC indicates the proportion of cases that can be dropped at random (creating a new partial network, with different centrality indices) while retaining with 95% certainty a 0.70 correlation with the order of the strength centralities indices in the original network. A CSC above 0.25 is recommended. A CSC of 0.50 or above is indicative of good robustness of the original network [57].

**Fig. 2:**
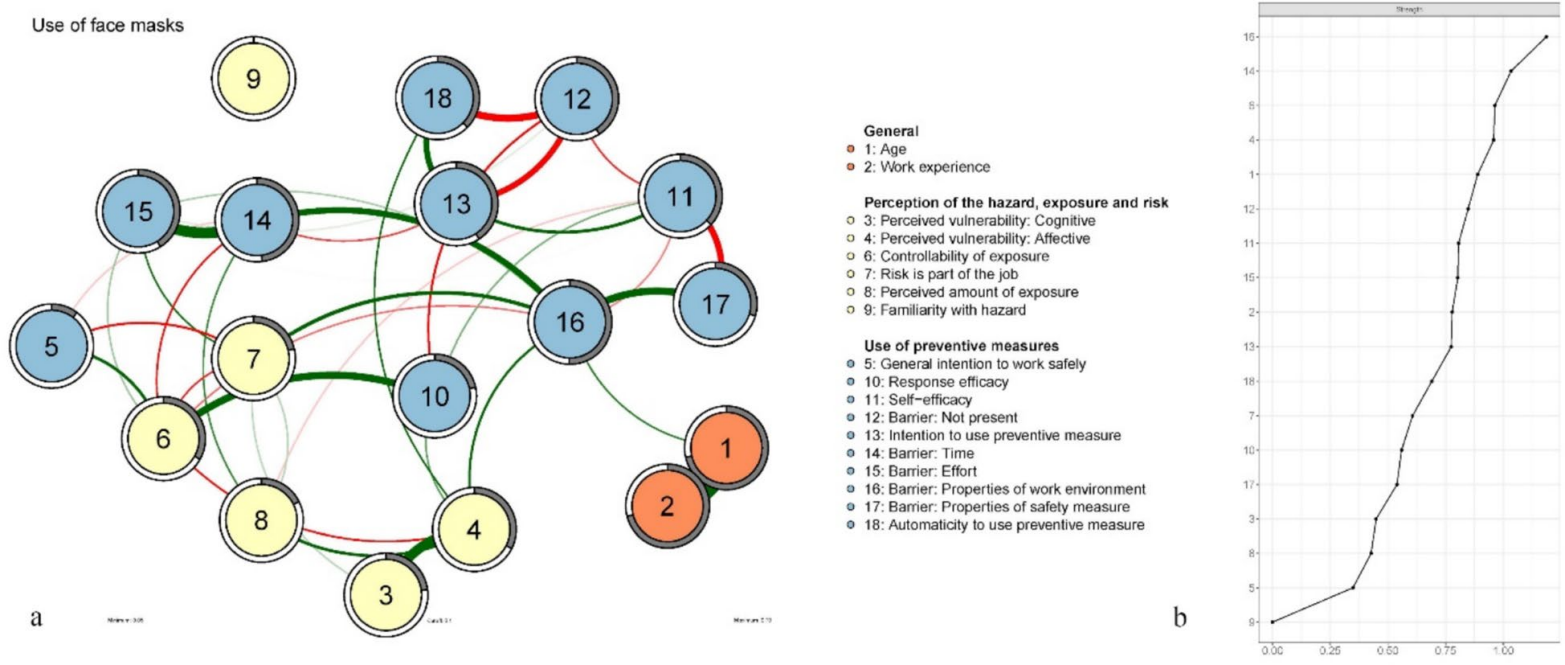
Face mask behavioural decision network (**a**) and strength centrality plots ordered by strength (**b**). Note. Colours indicate categorization as presented in Table 4

A Network Comparison Test (NCT) was not conducted to statistically test for differences between networks since the NCT has not been validated for within subjects data [59]. Networks are therefore only compared at a descriptive level. Networks were plotted using the *qgraph* (version 1.9.8) [60]. Network layout was plotted using the average layout for all three networks. A priori defined groups of variables were classified for the network visualization (see Fig. 2).

## Results

### Participants of main study

In total, 8 companies specialized in civil construction work (e.g. bridges and roadways) or housing construction agreed to contribute to the study. Questionnaire data was collected at 24 building sites and training locations. In total 187 questionnaires were included in this study. Characteristics of the sample population included in the main study are presented in Table 2.

**Table 2:** Characteristics of the sample population in the main study

Variable	
Sample size	187
Gender (% male)	100
Highest finished education (N, %)	
1 (low)	79 (42.2%)
2 (medium)	87 (46.5%)
3 (high)	12 (6.4%)
Age range (years)	17–63
Age mean (standard deviation)	46.2 (11.4)
Experience in construction work (years)	23.7 (12.5)

Education level according to CBS classification

Percentages highest finished education do not add up to 100 due to missing values (9)

### Means, standard deviations and non-parametric test results

Table 3 presents the means and standard deviations and non-parametric test results on drivers and barriers of using a face mask, dust collection on power tools, and using a vacuum cleaner instead of a broom (vacuuming). The table also includes an overview of non-parametric test results (Friedman test) of comparisons between drivers and barriers of using a face mask; dust collection on power tools; and using a vacuum cleaner instead of a broom (vacuuming).

**Table 3:** Means (M), standard deviations (SD) and non-parametric test results

Factors associated with the perception of the hazard, exposure, and risk					
Item	Valid responses	M (SD)			
Familiarity	187	2.95 (.97)			
Perceived amount of exposure	185	2.89 (1.01)			
Control over exposure	184	3.23 (1.19)			
General intention to work safely	186	4.02 (0.90)			
Perceived vulnerability (cognitive)	186	3.51 (.90)			
Perceived vulnerability (affective)	186	2.89 (1.07)			
Risk is part of the job	186	2.11 (1.04)			
Factors associated with the use of preventive measures					
	Valid responses	M(SD)			
		Face mask	Dust Collection	Vacuuming	Friedman test
Behavioural intention					
[…] I always use when I should	187; 187; 186	3.22 (1.08)	3.33 (1.06)	3.22 (1.01)	χ2(2) = 3.653, *p* = 0.161
Response efficacy					
[…] makes sure I don’t breathe in silica dust	186; 187; 187	3.51 (1.00)	3.36 (1.05)	3.63 (1.00)	χ2(2) = 9.966, *p* = 0.007
Self-efficacy					
[…] is easy to use	186; 187; 187	3.33 (1.05)	3.38 (1.04)	3.57 (1.01)	χ2(2) = 10.151, *p* = 0.006
Automaticity of use (scale)					
Scale total average	186;183;186	2.82 (0.81)	3.11 (.86)	2.97 (.86)	χ2(2) = 18.719, *p* < 0.001
Barriers^a^					
Preventive measure not present^R^	187; 187; 187	2.73 (1.08)	2.46 (1.02)	2.93 (1.16)	χ2(2) = 23.451, *p* < 0.001
Time	187; 187; 187	2.34 (.97)	2.55 (1.05)	2.58 (.96)	χ2(2) = 6.843, *p* = 0.033
Effort	186; 186; 187	2.20 (.93)	2.37 (.97)	2.40 (.93)	χ2(2) = 6.497, *p* = 0.039
Properties of work environment	187; 185; 187	2.31 (1.05)	2.79 (1.20)	2.75 (1.14)	χ2(2) = 20.728, *p* < 0.001
Properties of preventive measure	187; 187; 187	2.97 (1.18)	3.11 (1.20)	2.87 (1.09)	χ2(2) = 9.375, *p* = 0.009

Higher scores indicate stronger intentions, stronger beliefs on the respective items (e.g. stronger beliefs of vulnerability or stronger perceived barriers)

^a^Low scores indicate less agreement on the barrier being present

^R^Recoded

The Friedman test showed no significant differences in intention to use face masks, dust collection or vacuuming (χ2(2) = 3.653, *p* = 0.161). For all other drivers and barriers, statistically significant differences between preventive measures were found (see Table 3). Wilcoxon signed-rank test results of differences are further outlined below.

#### Response efficacy

The use of face masks is not considered more effective in preventing inhalation of silica dust than using dust collection on power tools (Z = −1.804, *p* = 0.071) or vacuuming (Z = −1.485, *p* = 0.137). Vacuuming is considered more effective than dust collection on power tools (Z = −3.086, *p* = 0.002).

#### Self-efficacy

Using a face mask is considered easier to do than vacuuming (Z = −2.781, *p* = 0.005) but not statistically significantly easier to do than using dust collection on power tools (Z = −0.852, *p* = 0.417). Using dust collection on power tools is considered easier to do than vacuuming (Z = −2.050, *p* = 0.040).

#### Automaticity of behaviour

Using dust collection on power tools is more automatically done than using face masks (Z = −3.394, *p* < 0.001) and vacuuming (Z = −2.555, *p* = 0.011). Vacuuming is more automatically done than using face masks (Z = −2.454, *p* = 0.014).

#### Barrier: on hand

Dust collection on power tools is more often on hand than a face mask (Z = −3.321, *p* < 0.001), and more often on hand than a vacuum (Z = −4.949, *p* < 0.001). A face mask is not more often on hand than a vacuum cleaner (Z = −1.867, *p* = 0.062).

#### Barrier: time

Using dust collection on power tools is more strongly considered to take too much time than using a face mask (Z = −2.109, *p* = 0.035), but not more than vacuuming (Z = −0.487, *p* = 0.627). Vacuuming is more strongly considered to take too much time to use than using a face mask (Z = −3.043, *p* = 0.002).

#### Barrier: effort benefit trade-off

Using dust collection on power tools is considered more purposeful than using a face mask (Z = −2.100, *p* = 0.036), but not less purposeful than vacuuming (Z = −0.558 *p* = 0.577). Vacuuming is considered more purposeful than using a face mask (Z = −2.363, *p* = 0.018).

#### Barrier: work environment

Using dust collection on power tools is more affected by the job that needs to be done than using a face mask (Z = −5.008, *p* < 0.001), but not more than vacuuming (Z = −0.569, *p* = 0.569). Vacuuming is more affected by the job that needs to be done than using a face (Z = −4.418, *p* < 0.001).

#### Barrier: complicates work

Using dust collection on power tools complicates work more than vacuuming (Z = −2.417, *p* < 0.016), but not more than using a face mask (Z = −1.627, *p* = 0.423). Vacuuming does not complicate work more than using a face mask (Z = −0.802, *p* = 0.423).

### Network analyses

**Fig. 3:**
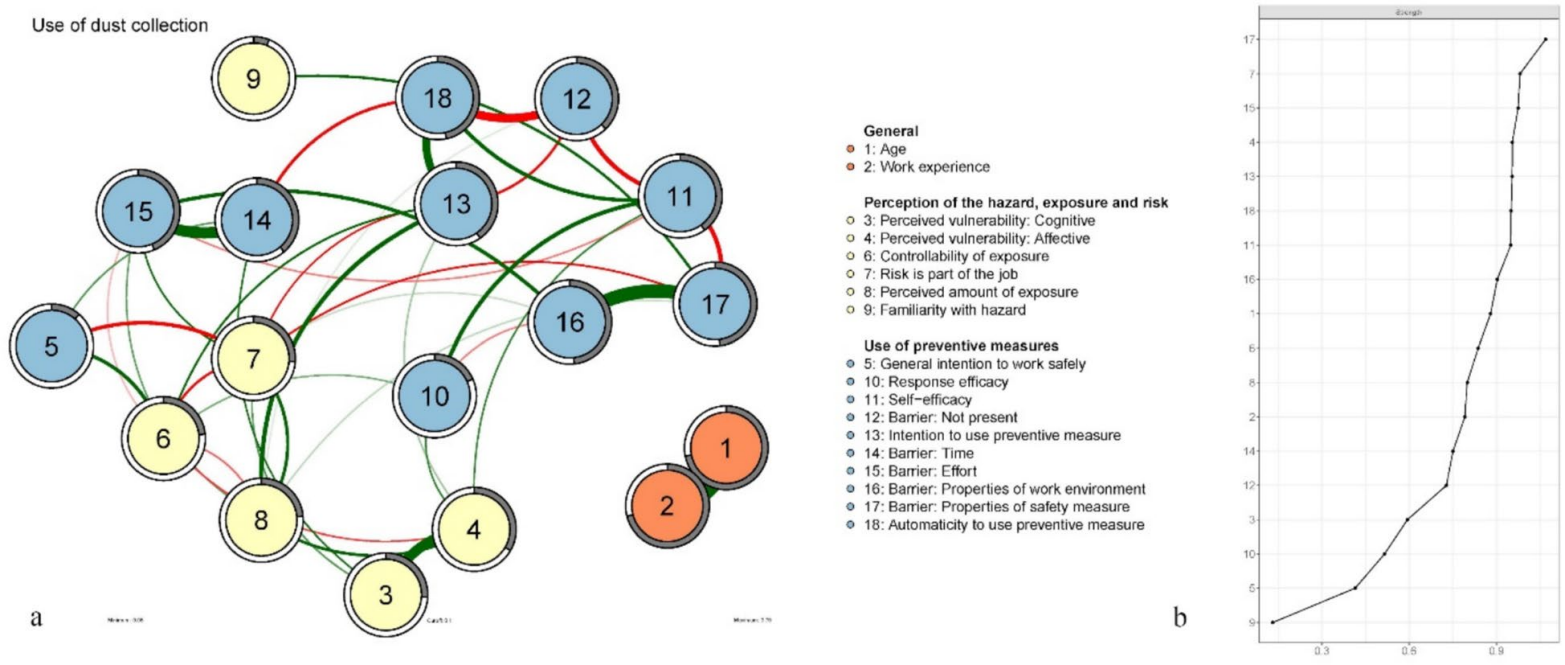
Dust Collection behavioural decision network (**a**) and strength centrality plots ordered by strength (**b**). Note. Colours indicate categorization as presented in Table 4

**Fig. 4:**
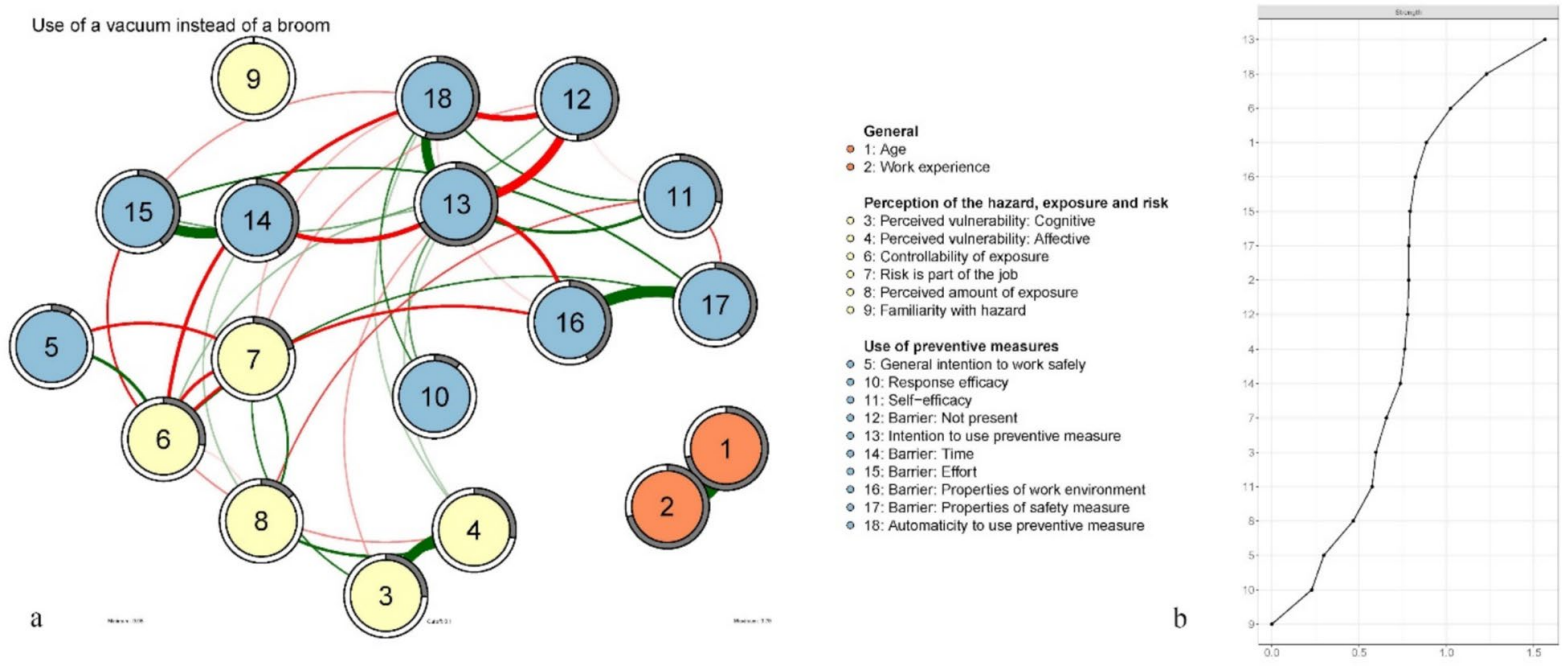
Vacuuming behavioural decision network (**a**) and strength centrality plots ordered by strength (**b**). Note. Colours indicate categorization as presented in Table 4

Figures 2a, 3a and 4a (Figures a) show the behavioural decision networks of using a face mask (FM, 2a), using dust collection on power tools (DC, 3a), and using a vacuum cleaner instead of a broom (VB, 4a). Recall that the actual use of face mask; dust collection on power tools; and a vacuum cleaner instead of a broom, are considered behaviours that are emergent of a (favourable) network topology. Strength centrality for each node (i.e. an estimation of how strongly a given node is connected to other nodes in the network), is presented in Figs. 2b, 3b and 4b (Figures b). Stability analysis of the strength centrality hierarchy showed good robustness for all networks (face mask CS-coefficient = 0.44; dust collection CS-coefficient = 0.52; vacuuming CS-coefficient = 0.67).

Table 4 present the results of the predictability analysis in terms of explained variance (R^2^). The predictability is depicted as a circle around each node in Figures a. The degree to which this circle is filled (grey) represents the proportion of variance in that node that can be explained by its connected neighbouring nodes. Predictability of nodes varies between networks. In all networks the nodes *age* and *work experience* are strongly associated with each other but disconnected from other nodes in the networks (see Figures a). Because age and work experience are logically dependent [56] we will not further elaborate on these variables.

**Table 4:** Explained variance (R^2^) of nodes in the face mask, dust collection and vacuuming networks

		R^2^			Range (R^2^)
		Face mask	Dust collection	Vacuuming	
Node					
General					
1	Age	.71	.72	.72	.71 –. 72
2	Work experience	.71	.71	.71	.71
Factors associated with the perception of the hazard, exposure, and risk					
9	Familiarity	.00	.06	.00	.00 –.06
6	Perceived exposure	.18	.24	.14	.14 –.24
8	Perceived control over exposure	.33	.23	.28	.23 –.33
5	General intention to work safely	.10	.11	.08	.08 –.10
3	Perceived vulnerability (cognitive)	.23	.26	.25	.23 –.26
4	Perceived vulnerability (affective)	.33	.33	.28	.28 –.33
7	Risk part of job	.21	.26	.21	.21 –.26
Factors associated with the use of face mask, dust collection or a vacuum cleaner					
13	Intention to use preventive measure	.41	.40	.67	.40 –.67
10	Response efficacy	.22	.18	.10	.10 –.22
11	Self-efficacy	.37	.40	.27	.27 –.40
18	Automaticity to use preventive measure	.38	.47	.55	.38 –.55
12	Barrier: Not present	.41	.38	.50	.38 –.50
14	Barrier: Time	.48	.39	.41	.39 –.48
15	Barrier: Effort	.41	.44	.40	.40 –.44
16	Barrier: Properties of work environment	.50	.48	.43	.43 –.50
17	Barrier: Properties of safety measure	.30	.48	.39	.30 –.48

#### Centrality

*Intention to use preventive measure* was the most central node in the VB network (1.56, R^2^ = 0.67). It has a moderate (5th node) position in the DC network (0.95, R^2^ = 0.40) and a moderate (10th node) position in the FM network (0.77, R^2^ = 0.41). Perceived barriers to using preventive measures play a relatively central role in all networks. Barriers *properties of work environment* (1.19, R^2^ = 0.50) and *time* (1.03, R^2^ = 0.48) are the most central nodes in the FM network. Barriers *properties of preventive measure* (1.07, R^2^ = 0.48) and *effort* (0.97, R^2^ = 0.44) are the 1 st and 3rd most central nodes in the DC network. The risk of getting ill from silica dust *being part of the job* is the second most central node in the DC network (1.07, R^2^ = 0.26), while only being the 12th node in the FM (0.61, R^2^ = 0.21) and VB (0.66, R^2^ = 0.21) networks. *Automaticity of using the preventive measure* is the 2nd most central node in the VB network (1.23, R^2^ = 0.55), while being the 6th in the DC network (0.95, R^2^ = 0.47) and 11th in the FM network (0.69, R^2^ = 0.37) Perceived *controllability of exposure* is the third most central node in the networks of using a FM (0.96, R^2^ = 0.33) and VB (1.02, R^2^ = 0.27). It is a moderately central node (10th node) in the DC network (0,84, R^2^ = 0.23). *Perceived vulnerability: Affective* (worry), holds a more central position than perceived vulnerability: cognitive (probability) in all networks, being the 4th node in the FM and DC networks (0.96, R^2^ = 0.33, and 0.95, R^2^ = 0.33 respectively) and the 10th node in the VB network (0.76, R^2^ = 0.28). *Perceived vulnerability: cognitive* (probability) is the 15th node in the FM (0.45, R^2^ = 0.23) and DC (0.59, R^2^ = 0.26) networks and the 13th in the VB (0.60, R^2^ = 0.25) network Perceived vulnerability (both cognitive and affective) is mostly indirectly and distantly associated with the intention to use preventive measures (see Figures a). The *general intention to work safely* is the second-to-last node in the FM (0.35, R^2^ = 0.10) network and the DC (0.41, R^2^ = 0.11) network. It is third-to-last in the VB network (0.30, R^2^ = 0.08) *Perceived familiarity with the hazard* was the least central node in face mask (0.00, R^2^ = 0.00), dust collection (0.13, R^2^ = 0.06) and vacuuming (0.00, R^2^ = 0.00) networks.

#### Edges

The barriers *effort* and *time* have a direct, relatively strong, positive association in the FM (0.41), DC (0.42) and VB (0.39) networks. *Properties of the work environment* and *properties of preventive measure* also share a direct, relatively strong, positive relationship in the FM (0.29), DC (0.50) and VB (0.43) networks. In all networks *barrier: not present* has a direct negative association with the *intention to use the preventive measure* (FM = −0.24, DC = −0.13, VB = −0.34)., and the *automaticity to use preventive measure* (FM = −0.28, DC = −0.33, VB = −0.23). Also in all networks, *automaticity* has a direct positive association with the *intention to use the preventive measures* (FM = −0.24, DC = −0.31, VB = −0.37). *Perceived vulnerability: affective* and *Perceived vulnerability: cognitive* share a direct, relatively strong, positive relationship in all networks (FM = 0.37, DC = 0.38, VB = 0.39).

## Discussion

The aim of this study was to present an explorative and descriptive account of risk perception, perceived barriers, and the use of three preventive measures aimed at reducing or preventing exposure to chemical substances in the construction industry. To do so, we outlined how construction workers perceive the hazard, exposure, and risk (e.g. familiarity or perceived exposure and worry) of silica dust. Also, we outlined perceived barriers, efficacy beliefs, and intentions regarding the use of face mask, dust collection or a vacuum cleaner (e.g. perceived effort, response efficacy and intention to use the preventive measure) to prevent exposure to silica dust. Finally, we applied a psychological network approach and studied the interdependence of all these factors to study potential intervention points to promote preventive behaviour with regard to occupational exposure to silica dust. Applying this approach provided several insights that can help foster the prevention and reduction of substance-related occupational diseases in construction. These insights are discussed in further detail below.

It is important to note that in this study and approach, safe behaviour is understood as an emergent property of a complex system of interacting variables (e.g. risk perceptions, efficacy beliefs, perceived barriers). Factors that are not directly associated with the intention to use preventive measures can thus still have a key role in the decision to use preventive measures. Our study hereby also provides an illustrative account of intention-behaviour gaps in the context of risk behaviours in construction [61].

### Risk perception of occupational exposure to silica dust

The current study showed that construction workers are generally inclined to belief there is a chance they will experience adverse health effects from exposure to silica dust during their work (perceived vulnerability – cognitive). At the same time, they are less likely to worry about this probability (perceived vulnerability – affective). In the behavioural decision networks this perceived vulnerability played a moderate role. Worry, as compared to the perceived chance, had a more central role, which indicates that concerns about the health risks likely play a more important role in the decision-making network than the perceived chance of experiencing health effects. Given that a lack of worry about the risks of exposure to silica dust can contribute to more unsafe behaviours [16, 17], these findings suggest that the current lack of concern about the personal risk of silica exposure may encourage unsafe behaviour.

In a qualitative study, Hambach and colleagues [25] concluded that workers often accept chemical risks associated with the job. However, in the current study construction workers mostly did not agree with the notion that the risks associated with exposure to silica dust are “part of the job.” While we did not specifically ask for acceptance (the item in the questionnaire read “*The fact you can get sick from silica dust is part of my job”*) this finding seems to contrast the qualitative findings by Hambach and colleagues [25] and the qualitative findings of our qualitative study. Furthermore, in the different decision networks the importance of this belief varied. That is, the belief played one of the most central roles (based on many weaker edges to other factors in the network) in the decision network of using dust collection on power tools. In this network, only the perceived barrier that the use of dust collection on power tools complicates work itself held a more central role (having a strong edge with the perceived barrier that using dust collection on power tools complicates the work itself). In the other networks it held a more moderate role.

Psychometric studies of risk perceptions have shown that familiarity with a hazard and controllability of exposure can affect perceptions of severity [34] and may therefore affect motivation for preventive behaviour [6]. Results of the current study also suggest that familiarity with silica dust hardly contributes little in the decision networks of using preventive measures and thus appears to have limited impact on the use of preventive behaviour. In line with the psychometric studies, the current study showed that constructions workers are likely to agree they can control exposure to silica dust. Furthermore, the network analyses showed that in the decision networks of using a face mask and using a vacuum cleaner instead of a broom, controllability was one of the most central factors. Therefore, the current study suggests that emphasizing control construction workers have on exposure to silica dust may indeed positively affect the use of preventive measures (cf. [6]).

### Perceived barriers to working safely with silica dust

The barriers to working safely included in this study were brought forward by construction workers in the exploratory qualitative interviews and therefore hold strong ecological validity. Study results indicate that similar barriers (e.g. *time* vs. *effort*, and *working environment complicating work* vs. *preventive measure complicating work*) are linked most strongly. Partial (but incomplete) explained variance indicates that these perceived barriers overlap but also play a unique role in the decision network. For example, barriers *time* and *effort* are relatively strongly linked in the behavioural decision networks, but variance in each variable is only partly explained, suggesting they may represent distinct intervention points. Importantly, all barriers (while in different orders of relative importance) play a key role in the behavioural decision networks. Therefore, in order to foster the use of preventive measures, efforts should be taken to lower these perceived barriers.

Some barriers may not easily be addressed by behavioural interventions. For example, carpenters may not be able to use dust collection on power tools when they have to drill holes in tight spots (i.e. the properties of the work environment). Others do present promising points of departure. For example, in all behavioural decision networks, not having the preventive measures at hand, is directly negatively associated with automaticity to use preventive measures, and the intention to use preventive measures. To overcome this barrier, efforts can be made to offer the measure close to the location where relevant tasks (e.g. drilling in concrete) are performed. For example, construction workers could be encouraged to carry a face mask in high exposure environments or for high exposure tasks (provided this does not obstruct them in any way). Similarly, dust collection for power tools, when not installed by default (or taken off by workers), should preferably be installed by default (see e.g., [62]), and should otherwise be offered at specific times and places when and where construction workers frequently work with silica containing materials. In the context of using a vacuum cleaner instead of a broom, efforts could be made to offer a vacuum cleaner at specific times when construction workers clean work sites. This will reduce time and effort in terms of walking to a specific distribution point. It will of course not be able to reduce the extra effort (and extra time) of using the vacuum cleaner. Offering the preventive measures at times and places when it is relevant for choice and behaviour of the construction worker likely also lowers the barriers ‘time’ and ‘effort.’

### Intention to work safely with silica dust

The current study provided several other interesting insights into intentions to work safely. First, results showed that constructions workers generally agreed they try to avoid exposure as much as possible (i.e. general intention to work safely). In contrast, they did not necessarily agree that they always use preventive measures when they should. This conflicting result holds for all three preventive measures. Interestingly, further analyses showed that general intention to work safely played little role in the behavioural decision networks, while specific intention to use preventive measures played a far more prominent role.

Construction companies frequently use mottos like “work safely or do not work at all” to emphasize the importance of working safely. While this motto establishes an important overarching value and long-term goal (also important in promoting work safety), findings of the current study suggest that promoting this general intention in risk communication strategies is unlikely to contribute to construction workers’ use of preventive measures. To promote specific behaviours that contribute to these long-term goals, risk communication efforts should therefore also address the specific intention to use preventive measures. These findings and interpretations are in line with Goal Setting Theory and the Construal Level Theory (CLT) of psychological distance, which state that setting specific short-term goals (e.g. I am taking a face mask with me tomorrow) are more effective for behavioural change than vague, abstract distant goals (e.g. I am going to avoid silica dust as much as possible) (see e.g., [63, 64]).

Results of the current study also showed that fostering automaticity with which construction workers use preventive measures can also contribute to safe behaviour by construction workers (cf. [65]). That is, construction workers’ level of automaticity to use face masks, dust collection and a vacuum cleaner instead of a broom was relatively low (all below the scale average). At the same time network analyses showed that automaticity to use preventive measures played a key role in the behavioural decision networks of using a vacuum cleaner instead of a broom and using dust collection on power tools. In all cases, automaticity had a strong direct positive link with the intention to use the preventive measure (see also explained variance). This suggest that improving automaticity may contribute to the use of preventive measures, both directly through increasing intention to use the measure, and as part of the behavioural decision network as a whole. Improving automaticity to use preventive measures, as part van (new) habit formation, is a slow process [66]. Frequency of behaviour, satisfaction, monitoring and rewarding can be important factors in habit formation [65, 66]. Reducing time and effort to use preventive measures (where possible) may boost the frequency with which workers preventive measures. Secondly, assuring positive affect or satisfaction with using preventive measures can help. For example, by making salient what amount of dust has not been inhaled due to using preventive measures such as face masks. Thirdly, monitoring and rewarding (i.e. positive reinforcement) consistent use of specific preventive measures over time. For example, site supervisors (or other designated persons) could randomly walk around a building site at times during the day and monitor use of preventive measures among individuals. Consistent use could be rewarded in some form.

Finally, results from all network analyses showed that neither age nor work experience played a prominent role in the behavioural decision networks. While the centrality measures for both variables were high, this can be explained by the logical dependence of the nodes [56]. Relationships with other nodes of the networks were mostly missing. This suggests that neither age nor work experience is a promising target for interventions aimed at improving preventive behaviour in the context of occupational exposure to silica dust.

### Strengths and limitations

A strength of this study is its complex systems (psychological network) approach. To our knowledge this study is the first application of the psychological network to study safe behaviour in the construction industry using a complexity perspective. This approach makes it possible to illustrate that (and: how) construction workers risk perceptions, decisions, and behaviours regarding occupational exposure to silica dust are part of a complex system of interacting (psychological) variables. Another strength of this study is that it builds on a qualitative pre study, in which relevant themes in construction workers decisions and behaviour were brought forward by the construction workers themselves. This qualitative foundation of a psychological network analysis strengthens the ecological validity and relevance of the quantitative results of the study.

A limitation of this study is the lack of a statistical test to compare the (within subjects) behavioural decision networks. However, the Network Comparison Test [67], used to compare psychological networks, has not been validated for within-subjects data. For this reason, we chose to omit these test results. Instead, we relied on a descriptive comparison. It should be noted that the application of psychological network analysis to the domain of risk perception research is still under development. Methodology needs to be developed further to substantiate findings using this approach.

The relatively small sample size for network analysis could be considered another limitation of the study. However, data met the analysis requirements and stability analysis indicated sufficiently robust results. We tried to include workers from small and medium-sized enterprises (SME’s) who served as subcontractors at the visited construction sites in our sample. Unfortunately, many of these workers decided not to participate after being informed that participation was voluntary. When interpreting the study results, it is therefore important to consider that the majority of participants were employed by large construction companies that devote considerable time to occupational health and safety. This may have introduced bias in the findings. It would be interesting to see if the structure of the behavioural decision networks holds for construction workers from SME’s and self-employed construction workers.

Finally, what may be considered a limitation of the study are the undirected associations between study variables. The explorative nature of this study should be taken into account in the interpretation of the results, especially in terms of possible target factors (i.e. nodes) for interventions to improve safe working practices.

## Conclusion

The current study addressed a need for practical knowledge to help prevent and reduce occupational diseases from exposure to hazardous substances. We did so by providing several possible points of departure to foster the use of preventive measures by construction workers when exposed to silica dust. We investigated how risk perception, perceived barriers to working safely, and intentions to work safely were related to the use of specific preventive measures. Secondly, we aimed to investigate whether differences can observed between distinct types of preventive measures. We described several differences in the behavioural decision networks for using a face mask, using dust collection on power tools, and using a vacuum cleaner instead of a broom. Increasing levels of concern, improving automaticity of use, addressing specific intention to use preventive measures in risk communication, and offering preventive measures at time and location where relevant tasks are performed, were discussed as possible points of departure to foster working safely with silica dust in construction. Future studies should further substantiate current exploratory findings.

## Supplementary Information

Supplementary Material 1.

Supplementary Material 2.

## Data Availability

The datasets used and/or analysed during the current study are available from the corresponding author on reasonable request and will be made publicly available after finalizing a second manuscript related to the dataset.
