# Supplementary material for: Risk perception, barriers, and working safely with silica dust in construction: a psychological network approach
Source: BMC Public Health. 2025 Jul 3;25:2318. doi: 10.1186/s12889-025-23347-2 (PMC12224377; doi:10.1186/s12889-025-23347-2)
Supplement: Supplementary file 1 — Supplementary Material 1. [file 12889_2025_23347_MOESM1_ESM.docx]

**Supplementary Material 1.** Questionnaire constructs, number of items and (example) items

| **Factor** | | **Items** | **(Example) item** |
| --- | --- | --- | --- |
|  | **General** | | |
| Age | | 1 | How old are you? |
| Work experience | | 1 | How many years have you been working in construction? |
|  | **Factors associated with the perception of the hazard, exposure and risk** | | |
| Familiarity | | 2 | “Working with silica dust is normal to me” |
| Perceived amount of exposure | | 1 | “During my work I inhale a lot of silica dust” |
| Control over exposure | | 1 | “I have control over whether I inhale silica dust or not” |
| General intention to work safely | | 1 | “I try to inhale as little silica dust as possible” |
| Perceived vulnerability: Cognitive | | 1 | “There is a probability I will get sick due to exposure to silica dust” |
| Perceived vulnerability: Affective | | 1 | “I worry about the possibility I can get sick due to exposure to silica dust” |
| Risk is part of the job | | 1 | “The fact you can get sick from silica dust is part of my job” |
|  | **Factors associated with the use of preventive measures** | | |
| Intention to use preventive measures | | 1 | “I *always* use dust collection on power tools when I should”  (emphasis in the questionnaire) |
| Self-efficacy | | 1 | “Vacuuming instead of brooming is easy to do”^a^ |
| Response efficacy | | 1 | “Using a face mask ensures I do not inhale silica dust”^a^ |
| Automaticity to use preventive measures | | 3 | “[…] I do without thinking about it” ^a^ |
| Barrier: Have on hand | | 1 | “[…] I have on hand when I need it” ^a , b^ |
| Barrier: Complicates work | | 1 | “[…] sometimes complicates my work” ^a , c^ |
| Barrier: Time | | 1 | “[…] takes too much time to use” ^a , c^ |
| Barrier: Work environment | | 1 | “[…] I sometimes cannot use because of my work” ^a , c^ |
| Barrier: Effort | | 1 | “[…] takes more effort than it yields” ^a , c^ |

a. Items were asked separately for using a face mask, using dust collection on power equipment, and using a vacuum cleaner instead of a broom.

b. Note that lower score indicate experiencing the barrier.

c. Note that higher score indicate experiencing the barrier.
