# Supplementary material for: Risk perception, barriers, and working safely with silica dust in construction: a psychological network approach
Source: BMC Public Health. 2025 Jul 3;25:2318. doi: 10.1186/s12889-025-23347-2 (PMC12224377; doi:10.1186/s12889-025-23347-2)
Supplement: Supplementary file 2 — Supplementary Material 2. [file 12889_2025_23347_MOESM2_ESM.docx]

#### ==============================================

#### STEP 0 INSTALL & LOAD R PACKAGES (check ROAM Research)

#### ==============================================

if (!require(Hmisc)) {

install.packages("Hmisc")}

if (!require(openxlsx)) {

install.packages("openxlsx")}

if (!require(mice)) {

install.packages("mice")}

if (!require(rtools)) {

install.packages("rtools")}

if (!require(dplyr)) {

install.packages("dplyr")}

if (!require(tidyverse)) {

install.packages("tidyverse")}

if (!require(RColorBrewer)) {

install.packages("RColorBrewer")}

if (!require(psychonetrics)) {

install.packages("psychonetrics")}

if (!require(bootnet)) {

install.packages("bootnet")}

if (!require(NetworkComparisonTest)) {

install.packages("NetworkComparisonTest")}

if (!require(mgm)) {

install.packages("mgm")}

if (!require(psych)) {

install.packages("psych")}

if (!require(igraph)) {

install.packages("igraph")}

if (!require(qgraph)) {

install.packages("qgraph")}

#### =======================================

#### STEP 1 SET WORKING DIRECTORY AND IMPORT DATA

#### =====================================

setwd("C:/Users/jansento/OneDrive - rivm.nl/Desktop/PREFER")

data_raw <- read.csv(file="PREFER_data file_NETWORK ANALYSIS.csv",

sep=",", #indicates how each value in the data file is separated

header=TRUE) #logical variable to indicate whether the data file contains headers

data_raw <- as.data.frame(data_raw)

## -------------------------------------------------

## INSPECT DATA

## -------------------------------------------------

# head(data_raw) #show first rows of data

# names(data_raw) # retrieve column name (column = variable)

# class(data_raw$"Bel_hazard") # Check the data type of a specific column

# dim(data_raw) # shows the dimension of the data (rows first, columns second)

# view(data_raw)

## ----------------------

## RECODE [...]_bar_onhand (was framed positively, while other barriers were framed negatively)

## ---------------------

data_raw <- data_raw %>%

mutate(FM_bar_onhand = recode(FM_bar_onhand, `5` = 1, `4` = 2, `3` = 3, `2` = 4, `1` = 5))

data_raw <- data_raw %>%

mutate(DC_bar_onhand = recode(DC_bar_onhand, `5` = 1, `4` = 2, `3` = 3, `2` = 4, `1` = 5))

data_raw <- data_raw %>%

mutate(VS_bar_onhand = recode(VS_bar_onhand, `5` = 1, `4` = 2, `3` = 3, `2` = 4, `1` = 5))

## -------------------------------------------------

## SELECT SILICA DATA from full data set.

## -------------------------------------------------

data = data_raw %>%

filter(Hazard == '1')

data = subset(data, select = -c(EP_resp_eff, EP_self_eff, EP_bar_onhand, EP_behavint, EP_bar_time, EP_bar_workar, EP_bar_comp, EP_Auto_total,

FH_resp_eff, FH_self_eff, FH_bar_onhand, FH_behavint, FH_bar_time, FH_bar_effort, FH_bar_env, FH_bar_comp, FH_Auto_total))

## --------------------------------------

## General Descriptives

## --------------------------------------

# head(data)

# str(data)

# names(data)

# summary(data)

mean(data$Familiarity_total,na.rm = TRUE) # 2.95

sd(data$Familiarity_total, na.rm = TRUE) # .97

mean(data$Exp_perc_amount,na.rm = TRUE) # 2.89

sd(data$Exp_perc_amount, na.rm = TRUE) # 1.01

mean(data$Control_personal,na.rm = TRUE) # 3.23

sd(data$Control_personal, na.rm = TRUE) # 1.19

mean(data$Gen_behavint,na.rm = TRUE) # 4.02

sd(data$Gen_behavint, na.rm = TRUE) # .90

mean(data$Risk_perc_vul,na.rm = TRUE) # 3.51

sd(data$Risk_perc_vul, na.rm = TRUE) # .90

mean(data$Risk_perc_wor,na.rm = TRUE) # 2.89

sd(data$Risk_perc_wor, na.rm = TRUE) # 1.07

mean(data$Risk_Accept_partofjob,na.rm = TRUE) # 2.11

sd(data$Risk_Accept_partofjob, na.rm = TRUE) # 1.04

mean(data$FM_bar_onhand,na.rm = TRUE) # 2.73 (recoded)

sd(data$FM_bar_onhand, na.rm = TRUE) # 1.08

mean(data$DC_bar_onhand,na.rm = TRUE) # 2.46 (recoded)

sd(data$DC_bar_onhand, na.rm = TRUE) # 1.02

mean(data$VS_bar_onhand,na.rm = TRUE) # 2.93 (recoded)

sd(data$VS_bar_onhand, na.rm = TRUE) # 1.16

##------------------------------------------------------

## CREATE GROUPS (Theory/Qualitatively driven) for networks and legends (After)

##------------------------------------------------------

groups <- list(c(1,2), c(3,4,6,7,8,9), c(5,10,11,12,13,14,15,16,17,18))

names(groups) <- c("General",

"Perception of the hazard, exposure and risk",

"Use of preventive measures")

group_colors <- brewer.pal(3, "Set2")

## -----------------------------------------------------

## CHECK MISSING DATA from selected data

## -----------------------------------------------------

#check N/A's

summary(data) #check how many NAs are present per variable

# change from integer to numeric

data <- as.data.frame(sapply(data, as.numeric))

## -------------------------------------------------------

## IMPUTE MISSING DATA

## -------------------------------------------------------

# mean impute missing data full PPE file

Imput_data <- data %>%

mutate_all( ~replace_na(., mean(., na.rm = TRUE)))

#Check NAs

# summary(Imput_data)

# view(Imput_data)

### -------------------------------------------------------

### Select imputed data for PPE's

### -------------------------------------------------------

#FACE MASK

data_FM <- Imput_data %>%

select(c(Age,

Work_experience,

Risk_perc_vul,

Risk_perc_wor,

Gen_behavint,

Control_personal,

Risk_Accept_partofjob,

Exp_perc_amount,

Familiarity_total,

starts_with("FM")))

# DUST COLLECTION

data_DC <- Imput_data %>%

select(c(Age,

Work_experience,

Risk_perc_vul,

Risk_perc_wor,

Gen_behavint,

Control_personal,

Risk_Accept_partofjob,

Exp_perc_amount,

Familiarity_total,

starts_with("DC")))

# VACUUMING

data_VS <- Imput_data %>%

select(c(Age,

Work_experience,

Risk_perc_vul,

Risk_perc_wor,

Gen_behavint,

Control_personal,

Risk_Accept_partofjob,

Exp_perc_amount,

Familiarity_total,

starts_with("VS")))

## ------------------------------------------------------

## Set node names for reference

## ------------------------------------------------------

nNames <- c(

"1", #age

"2", #work experience

"3", # Vulnerability (chance) | Risk perception chance

"4", # Vulnerability (worry) | Risk perception worry

"5", # General Intent

"6", # Controllability

"7", # Risk acceptance | Part of job ## -> check label

"8", # Peceived exposure

"9", # Familiarity

"10", # Response efficacy

"11", # Self-efficacy

"12", # Barrier: on hand

"13", # Specfic intent

"14", # Barrier: Time

"15", # Barrier: Effort

"16", # Barrier: Work conditions

"17", # Barrier: Complicates

"18" # Automaticity of use

)

lNames <- c(

"Age",

"Work experience",

"Perceived vulnerability: Cognitive",

"Perceived vulnerability: Affective",

"General intention to work safely",

"Controllability of exposure",

"Risk is part of the job",

"Perceived amount of exposure",

"Familiarity with hazard",

"Response efficacy",

"Self-efficacy",

"Barrier: Not present",

"Intention to use preventive measure",

"Barrier: Time",

"Barrier: Effort",

"Barrier: Properties of work environment",

"Barrier: Properties of preventive measure",

"Automaticity to use preventive measure"

)

lNames_numbers <- c(

"1",

"2",

"3",

"4",

"5",

"6",

"7",

"8",

"9",

"10",

"11",

"12",

"13",

"14",

"15",

"16",

"17",

"18"

)

## ---------------------------------------------

## ESTIMATE MGM

## Mean imputation applied to N/A

## ---------------------------------------------

# FACE MASK

#convert df to matrix to accomodate mgm

data_FM_matrix <- as.matrix(data_FM)

set.seed(1)

data_FM_mgm <- mgm(data = data_FM_matrix,

type = rep("g", ncol(data_FM_matrix)),

levels = rep(1, ncol(data_FM_matrix)),

k = 2,

lambdaSel = "CV", #regularization = cross validation (less conservative)

ruleReg = "AND",

threshold = "none")

# DUST COLLECTION

#convert df to matrix to accomodate mgm

data_DC_matrix <- as.matrix(data_DC)

set.seed(1)

data_DC_mgm <- mgm(data = data_DC_matrix,

type = rep("g", ncol(data_DC_matrix)),

levels = rep(1, ncol(data_DC_matrix)),

k = 2,

lambdaSel = "CV", #regularization = cross validation (less conservative)

ruleReg = "AND",

threshold = "none")

# VACUUMING

#convert df to matrix to accomodate mgm

data_VS_matrix <- as.matrix(data_VS)

set.seed(1)

data_VS_mgm <- mgm(data = data_VS_matrix,

type = rep("g", ncol(data_VS_matrix)),

levels = rep(1, ncol(data_VS_matrix)),

k = 2,

lambdaSel = "CV", #regularization = cross validation (less conservative)

ruleReg = "AND",

threshold = "none")

?ruleReg

## ----------------------------------------------

## CREATE AVERAGE LAYOUT AND MAX VALUE

## ----------------------------------------------

# create average network layout PPE silica

net_layout <- averageLayout(data_FM_mgm$pairwise$wadj,

data_DC_mgm$pairwise$wadj,

data_VS_mgm$pairwise$wadj,

layout = "spring")

#create max_val for PPE silica networks

max_val <- max(max(data_FM_mgm$pairwise$wadj),

max(data_DC_mgm$pairwise$wadj),

max(data_VS_mgm$pairwise$wadj))

## ----------------------------------------------

## ESTIMATE predictability

## ----------------------------------------------

pred_FM <- predict(object = data_FM_mgm,

data = data_FM,

errorCon = 'R2')

pred_DC <- predict(object = data_DC_mgm,

data = data_DC,

errorCon = 'R2')

pred_VS <- predict(object = data_VS_mgm,

data = data_VS,

errorCon = 'R2')

pred_FM$errors #R2 for variables in Face masks network

pred_DC$errors

pred_VS$errors

?predict.mgm

## ========================================================================

## VISUALIZE MGM NETWORKS

## Edges between Gaussian variables can be considered partial correlations

## These figures are presented in the paper

## ========================================================================

group_colors <- brewer.pal(3, "RdYlBu")

# FACE MASKS

graph_FM <- qgraph(data_FM_mgm$pairwise$wadj, # weighted adjacency matrix as input

layout = net_layout,

maximum = max_val,

title.cex = 2.00,

legend = TRUE,

legend.cex = 0.80,

layoutOffset = c(-0.15, 0),

groups = groups,

colors = group_colors,

pie = pred_FM$errors[,2], # provide errors as input

pieColor = rep('gray50'), #rep = repeat colour for all nodes

edge.color = data_FM_mgm$pairwise$edgecolor,

edge.labels = FALSE, # edge.label.cex=0.5,

labels = nNames, palette("ggplot2"),

nodeNames = lNames,

minimum = 0.05,

cut = 0.10,

curve = 1.0, curveAll = TRUE,

details = TRUE,

theme = "colorblind",

vsize=8, esize=15,

title = "Use of face masks")

# Extract centrality measures and edge weights in tables and create excel file

centrality_FM <- centrality_auto(graph_FM)

centrality_FM_df <- as.data.frame(centrality_FM$node.centrality)

edges_FM_df <- as.data.frame(graph_FM$Edgelist)

FM_workbook <- createWorkbook()

addWorksheet(FM_workbook, "Centrality Values")

addWorksheet(FM_workbook, "Edge weights")

writeData(FM_workbook, "Centrality Values", centrality_FM_df)

writeData(FM_workbook, "Edge weights", edges_FM_df)

saveWorkbook(FM_workbook, "FM centrality values and edge weights.xlsx", overwrite = TRUE)

#DUST COLLECTION

graph_DC <- qgraph( data_DC_mgm$pairwise$wadj, # weighted adjacency matrix as input

layout = net_layout,

maximum = max_val,

title.cex = 2.00,

legend = TRUE,

legend.cex = 0.80,

layoutOffset = c(-0.15, 0),

groups = groups,

colors = group_colors,

pie = pred_DC$errors[,2], # provide errors as input

pieColor = rep('gray50'), #rep = repeat colour for all nodes

edge.color = data_DC_mgm$pairwise$edgecolor,

edge.labels = FALSE, # edge.label.cex=0.5,

labels = nNames, palette("ggplot2"),

nodeNames = lNames,

minimum = 0.05,

cut = 0.10,

curve = 1.0, curveAll = TRUE,

details = TRUE,

theme = "colorblind",

vsize=8, esize=15,

title = "Use of dust collection")

# Extract centrality measures and edge weights in tables and create excel file

centrality_DC <- centrality_auto(graph_DC)

centrality_DC_df <- as.data.frame(centrality_DC$node.centrality)

edges_DC_df <- as.data.frame(graph_DC$Edgelist)

DC_workbook <- createWorkbook()

addWorksheet(DC_workbook, "Centrality Values")

addWorksheet(DC_workbook, "Edge weights")

writeData(DC_workbook, "Centrality Values", centrality_DC_df)

writeData(DC_workbook, "Edge weights", edges_DC_df)

saveWorkbook(DC_workbook, "DC centrality values and edge weights.xlsx", overwrite = TRUE)

# VACUUMING

graph_VS <- qgraph( data_VS_mgm$pairwise$wadj, # weighted adjacency matrix as input

layout = net_layout,

maximum = max_val,

title.cex = 2.00,

legend = TRUE,

legend.cex = 0.80,

layoutOffset = c(-0.15, 0),

groups = groups,

colors = group_colors,

pie = pred_VS$errors[,2], # provide errors as input

pieColor = rep('gray50'), #rep = repeat colour for all nodes

edge.color = data_VS_mgm$pairwise$edgecolor,

edge.labels = FALSE, # edge.label.cex=0.5,

labels = nNames, palette("ggplot2"),

nodeNames = lNames,

minimum = 0.05,

cut = 0.10,

curve = 1.0, curveAll = TRUE,

details = TRUE,

theme = "colorblind",

vsize=8, esize=15,

title = "Use of a vacuum instead of a broom")

# Extract centrality measures and edge weights in tables and create excel file

centrality_VS <- centrality_auto(graph_VS)

centrality_VS_df <- as.data.frame(centrality_VS$node.centrality)

edges_VS_df <- as.data.frame(graph_VS$Edgelist)

VS_workbook <- createWorkbook()

addWorksheet(VS_workbook, "Centrality Values")

addWorksheet(VS_workbook, "Edge weights")

writeData(VS_workbook, "Centrality Values", centrality_VS_df)

writeData(VS_workbook, "Edge weights", edges_VS_df)

saveWorkbook(VS_workbook, "VS centrality values and edge weights.xlsx", overwrite = TRUE)

pdf("New Graphs FM, DC, VS.pdf", height=12, width=22)

plot(graph_FM)

plot(graph_DC)

plot(graph_VS)

dev.off()

## =========================

## Centrality (Strength)

## =========================

Strength_FM <- centralityPlot(data_FM_mgm$pairwise$wadj,

include = ("Strength"),

scale = "raw",

orderBy = "Strength",

labels = lNames_numbers)

Strength_DC <- centralityPlot(data_DC_mgm$pairwise$wadj,

include = ("Strength"),

scale = "raw",

orderBy = "Strength",

labels = lNames_numbers)

Strength_VS <- centralityPlot(data_VS_mgm$pairwise$wadj,

include = ("Strength"),

scale = "raw",

orderBy = "Strength",

labels = lNames_numbers)

plot(Strength_DC)

# -------------------------

# Create PDF's

# -------------------------

pdf('Centrality Plot Face Mask.pdf', width = 6, height = 12)

Strength_FM + theme(axis.text = element_text(size = 14))

dev.off()

pdf('Centrality Plot Dust Collection.pdf', width = 6, height = 12)

Strength_DC + theme(axis.text = element_text(size = 14))

dev.off()

pdf('Centrality Plot Vacuuming.pdf', width = 6, height = 12)

Strength_VS + theme(axis.text = element_text(size = 14))

dev.off()

pdf('Figure X Combined Centrality plots.pdf')

centralityPlot(list("Face mask"=graph_FM,

"Dust collection"=graph_DC,

"Vacuuming"=graph_VS),

include = c("Strength"),

scale = "raw",

orderBy = "default")

dev.off()

## ========================================================

##

## Analyze stability of networks using Bootnet

##

## ========================================================

boot_FM <- bootnet(data_FM, default = "mgm",

statistics = c("strength", "edge", "expectedInfluence"),

nBoots = 1000,

type = "nonparametric",

nCores = 8)

boot_DC <- bootnet(data_DC, default = "mgm",

statistics = c("strength", "edge", "expectedInfluence"),

nBoots = 1000,

type = "nonparametric",

nCores = 8)

boot_VS <- bootnet(data_VS, default = "mgm",

statistics = c("strength", "edge", "expectedInfluence"),

nBoots = 1000,

type = "nonparametric",

nCores = 8)

boot_FM_case <- bootnet(data_FM, default = "mgm",

statistics = c("strength", "edge", "expectedInfluence"),

nBoots = 1000, type = "case",

nCores = 8)

boot_DC_case <- bootnet(data_DC, default = "mgm",

statistics = c("strength", "edge", "expectedInfluence"),

nBoots = 1000,

type = "case",

nCores = 8)

boot_VS_case <- bootnet(data_VS, default = "mgm",

statistics = c("strength", "edge", "expectedInfluence"),

nBoots = 1000,

type = "case",

nCores = 8)

?bootnet

pdf("Bootstrap results.pdf")

plot(boot_FM, labels = FALSE, order = "sample")

plot(boot_DC, labels = FALSE, order = "sample")

plot(boot_VS, labels = FALSE, order = "sample")

dev.off()

## ---------------------

## Plot differences in edge-strength (p = .05) (black = sig difference)

## ---------------------

pdf("Bootstrap results Edge difference (p=.05).pdf")

plot(boot_FM, "edge", plot = "difference", onlyNonZero = TRUE, order = "sample", title("Face mask"))

plot(boot_DC, "edge", plot = "difference", onlyNonZero = TRUE, order = "sample", title("Dust collection"))

plot(boot_VS, "edge", plot = "difference", onlyNonZero = TRUE, order = "sample", title("Vacuum"))

dev.off()

## ---------------------------

## Plot differences (p = .05) IN CENTRALITY (node strength) indices

## ---------------------------

pdf("Bootstrap results Strength difference (p=.05).pdf")

plot(boot_FM, "strength", plot = "difference", title("Face mask"))

plot(boot_DC, "strength", plot = "difference", title("Dust collection"))

plot(boot_VS, "strength", plot = "difference", title("Vacuum"))

dev.off()

##----------------------------------------

## Analyze stability of centrality indices

## CALCULATE CORRELATIONAL STABILITY COEFFICIENT (EPSKAMP et al., 2018)

##----------------------------------------

corStability(boot_FM_case) #CS-coefficient = 0.44 indicating good robustness

corStability(boot_DC_case) #CS-coefficient = 0.52 indicating good robustness

corStability(boot_VS_case) #CS-coefficient = 0.59 indicating good robustness
